# A case report of anterior mediastinal signet ring cell carcinoma

**DOI:** 10.1097/MD.0000000000032202

**Published:** 2022-12-02

**Authors:** Simin Liu, Anbang Zhao, Ming Mao

**Affiliations:** a Department of Immunology, Tongji Medical College, Huazhong University of Science & Technology, Wuhan, China; b Department of Hepatobiliary and Pancreatic Surgery, Zhongnan Hospital of Wuhan University, Wuhan, China; c Department of Thoracic Surgery, Zhongnan Hospital of Wuhan University, Wuhan, China.

**Keywords:** adenocarcinoma, mediastinal tumor, Signet ring cell carcinoma, thymic cancer

## Abstract

Anterior mediastinal signet ring cell carcinoma (SRCC) is a rare tumor that has only been reported in two cases of thymic cancer. Positive immunohistochemistry (IHC) staining for caudal-type homeobox (CDX) 2, cytokeratin (CK) 20 and special AT-rich binding protein (SATB) 2 usually indicate gastrointestinal tumors but begin to appear in thymic cancers with enteric differentiation. Here, we describe a case of the anterior mediastinal SRCC with enteric differentiation who was correctly treated with surgery and chemo-radiation and was alive after four months.

**Patient concerns:**

A 48-year-old female presented without chest and lung symptoms had an anterior mediastinal mass during a routine physical examination. Laboratory examinations showed an elevated level of serum carbohydrate antigen (CA)-125 at 73.63 U/mL. Chest computed tomography (CT) showed an irregular soft tissue density shadow with heterogeneous enhancement in the anterior mediastinum. The tumor had invaded the pericardium, the left septal nerve and the innominate and was completely removed after anterior mediastinal surgery. Postoperative pathological examinations revealed signet ring cell features and positive staining for CDX2, CK20, SATB2 and Ki-67 (Li: 70%). The samples were negative for cluster of differentiation (CD)-5, CK7, thyroid transcription factor (TTF) 1, NapsinA, CerbB-2, P53 and PD-L1 by IHC examinations. The suspected diagnosis was an anterior mediastinal SRCC that had originated in the digestive system.

**Diagnosis:**

The patient was diagnosed with anterior mediastinal SRCC.

**Interventions:**

The patient was treated with surgery and combined chemo-radiotherapy.

**Outcomes:**

The patient had no recurrence or metastasis after five months.

**Lessons:**

We describe a rare case of the anterior mediastinal SRCC of unknown origin. Our case, for the first time shows that surgery combined with chemo-radiotherapy is an effective treatment regimen for anterior mediastinal SRCC.

## 1. Introduction

Signet ring cell carcinoma (SRCC) is a form of adenocarcinoma that can secrete mucin and is characterized by high invasion and poor prognosis^[1–7]^ and can originate in several tissues such as the gastrointestinal tract, biliary tract, bladder and breast. The early diagnosis of SRCC remains challenging and involves histological or immuno-histochemical (IHC) examinations. SRCC shows cytoplasmic vacuolation and the nucleus may be shifted due to intracellular mucin.^[8]^ Previous studies have reported differential IHC reactivity in primary SRCC cases with cytokeratin (CK) 20, caudal-type homeobox (CDX) 2 and special AT-rich binding protein (SATB2) positive staining in primary gastrointestinal SRCC,^[9]^ and thyroid transcription factor (TTF)-1 and NapsinA positive staining in primary lung SRCC.^[10]^ Anterior mediastinal SRCC is rare and the first related case of a lymphoepithelial cystic lesion (LECL) dates back to 1989.^[11]^ Only 2 cases of primary thymic SRCC have been since been reported.^[12]^ Here, we report on a unique case of the anterior mediastinal SRCC.

## 2. Case report

**Figure 1.**
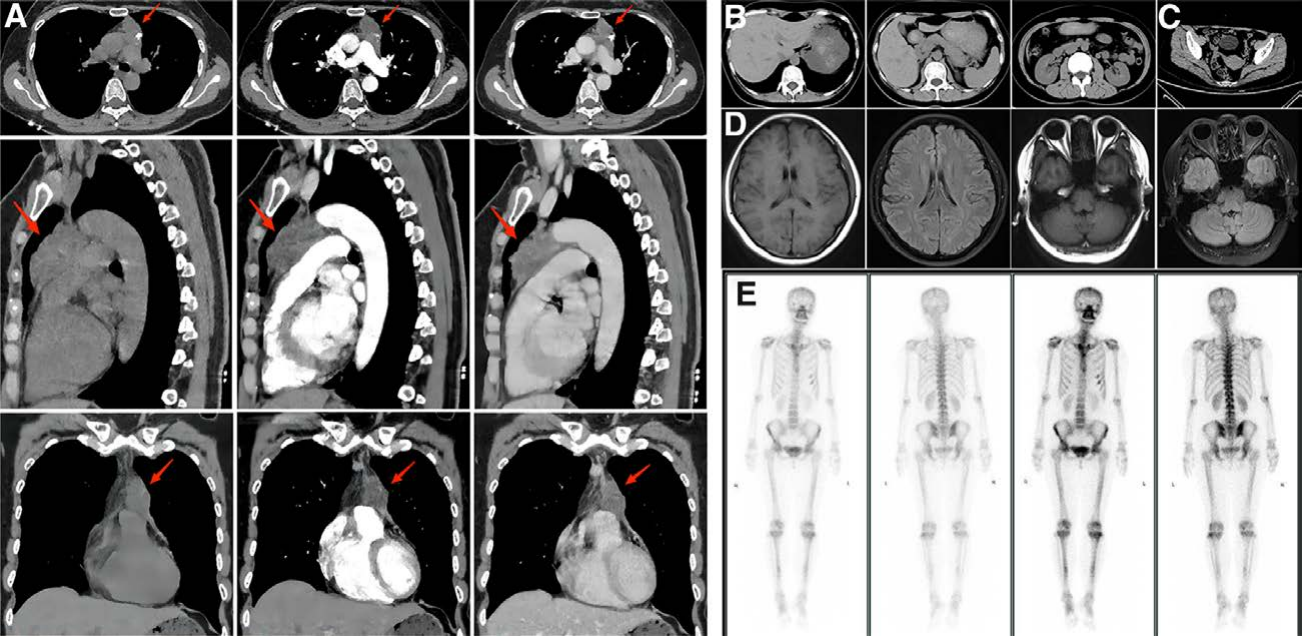
Radiological examination before surgery. (a) A huge irregular soft tissue density shadow (the red arrow) measuring 4.8 * 2.5 cm in size was observed in the anterior mediastinum by chest CT, with uneven enhancement in the arterial and venous phase. (b‐d) There was normal in abdominal CT, pelvic CT and brain MRI. (e) Whole-body bone ECT examination revealed that partial bone metabolism was slightly active, which indicated the benign disease.

A 48-year-old female with no history of smoking or alcohol abuse was admitted to Zhongnan Hospital of Wuhan University. Physical examinations identified an anterior mediastinal mass yet the patient did not have any chest or lung symptoms. Laboratory analysis showed an elevated level of the serum tumor marker carbohydrate antigen (CA)-125 at 73.63 U/mL (normal range 0‐35 U/mL), whilst other biomarkers including β-hCG, CA-199, CEA were normal. Chest computed tomography (CT) of the mediastinal window showed an irregular soft tissue density shadow measuring 4.8 × 2.5 cm. Enhancement of the mass was heterogeneous owing to its irregular density and calcification. The fat space between the lesion and the adjacent aorta and pulmonary artery disappeared (Fig. 1a). Abdominal CT, brain MRI, and whole-body bone Emission Computed Tomography (ECT) were acquired. Given the elevated level of CA-125, pelvic CT was also undertaken but no extrathoracic tumor lesions were observed (Fig. 1b‐e).

A mediastinal mass biopsy was recommended but the patient elected for surgery. The anterior mediastinal operation was performed on the 4th day of admission. The mass had invaded the pericardium and the left septal nerve. The innominate vein was 10 × 8 × 3 cm in size and irregularly shaped. The tumor was completely resected and the affected pericardium and innominate vein were removed. The remaining pericardium was drained by fenestration and repaired with a cardiac patch.

**Figure 2.**
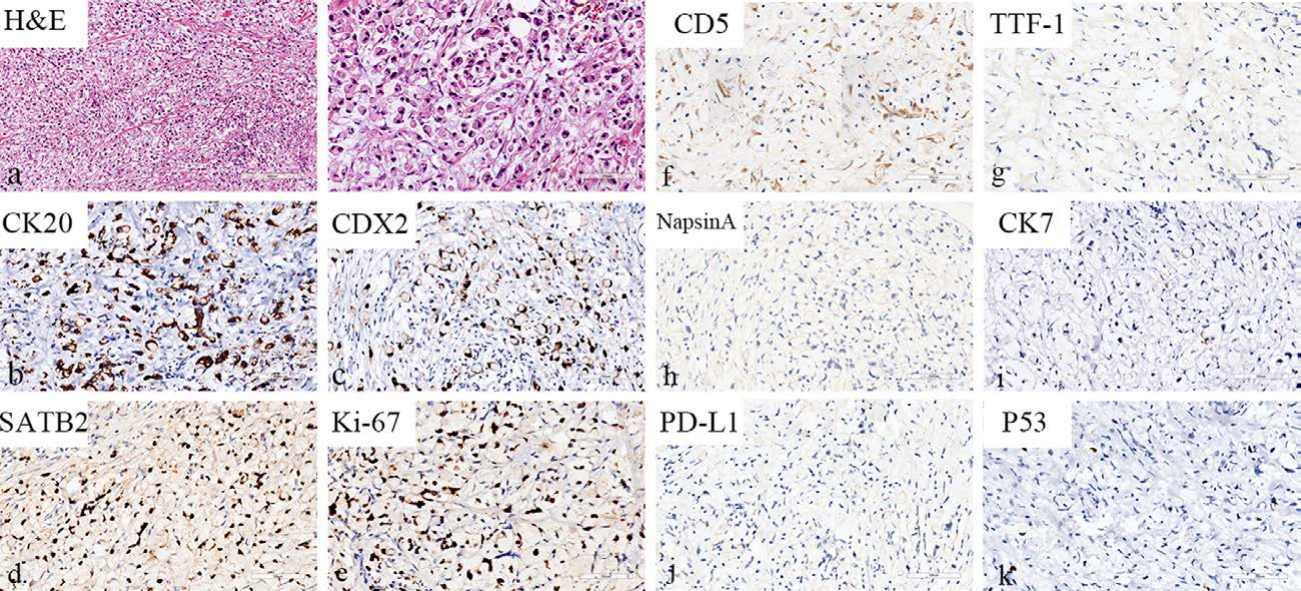
Pathological analysis of the anterior mediastinal mass. (a) H&E staining showed cytoplasm was vacuolated by intracellular mucins and the nucleus was pushed to one side, which was highly consistent with the morphology of signet ring cells. (b‐e) The tumor could be observed positive for CK20, CDX2, SATB2 and Ki-67 by IHC. (f‐k) Negative results of CD5, TTF-1, NapsinA, CK7, PD-L1 and P53 were shown through IHC.

**Figure 3.**
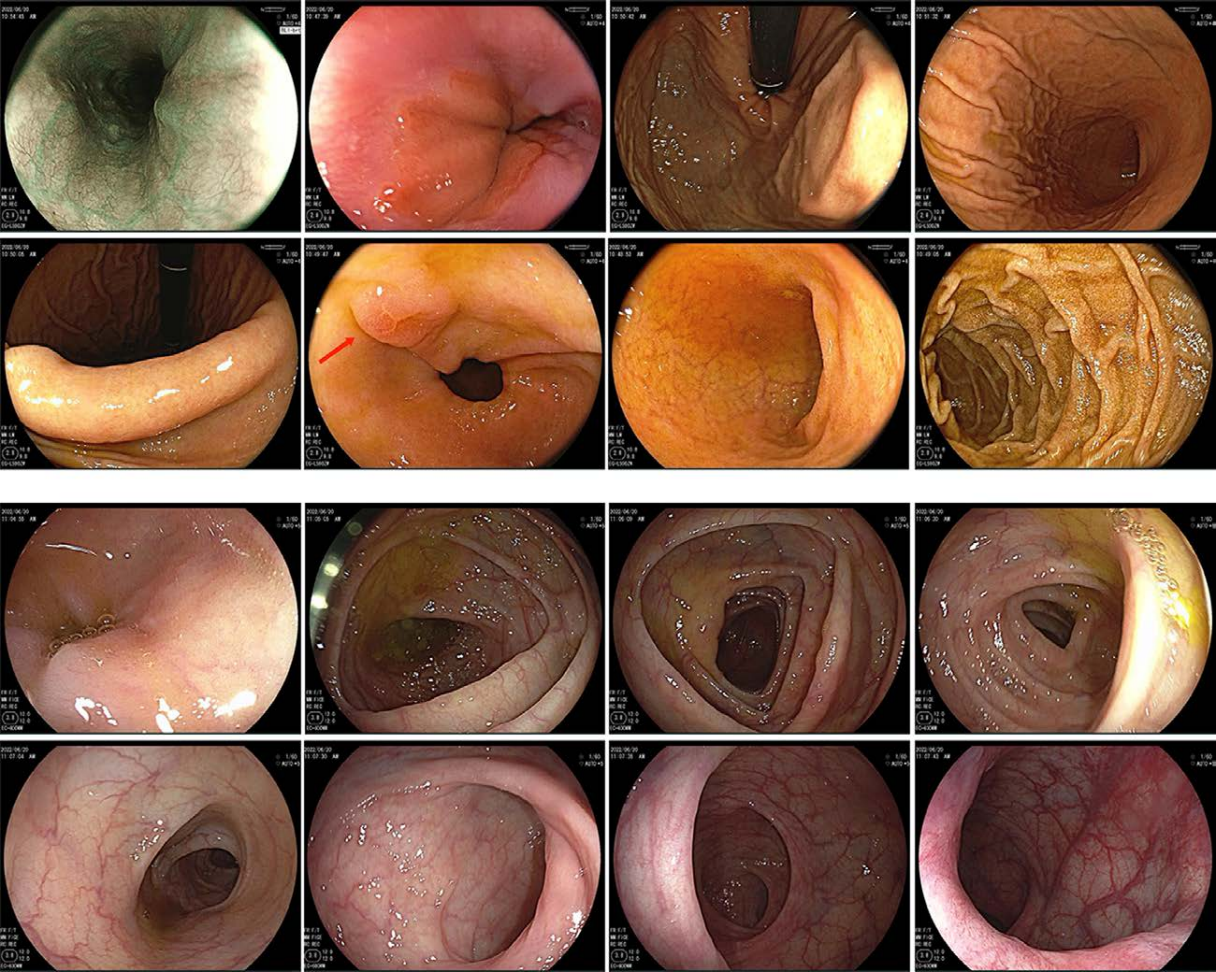
Gastroenteroscopy and colonoscopy after surgery. Gastrointestinal endoscopy and colonoscopy revealed that a flake of erosion which indicated intestinal metaplasia could be found in lesser curvature of the gastric antrum (the red arrow), and no abnormalities were found in other parts.

**Figure 4.**
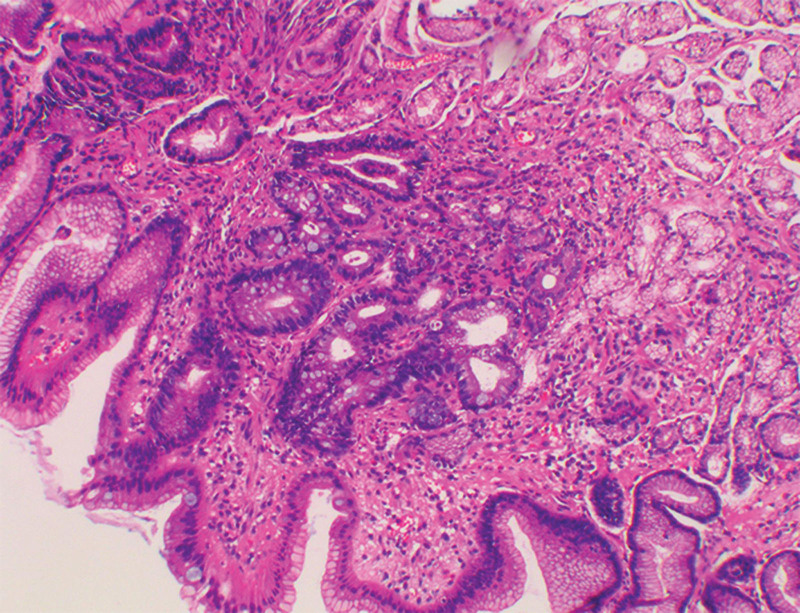
Pathological analysis of the gastric disease. H&E staining showed only mild chronic non-atrophic gastritis with mild intestinal metaplasia of glands could be observed.

Histopathological examinations revealed neoplastic cells filled with intracytoplasmic vacuoles that contained abundant mucins and the nuclei were pushed to one side. Cytological morphology suggested a diagnosis of SRCC. The IHC staining results were as followed: CDX2, SATB2, CK20 (+), Ki-67 (Li: 70%), cluster of differentiation (CD)5, CK7, TTF-1, NapsinA, CerbB-2, P53, EBER (-), PD-L1 (CPS = 0) (Fig. 2). Since the high expression of CDX2, SATB2, CK20 usually suggested tumors of gastrointestinal origin, the patient underwent gastroenteroscopy and colonoscopy on postoperative day (POD) 12 (Figs. 3 and 4). Mild, chronic, non-atrophic gastritis with mild intestinal metaplasia of glands was observed and IHC staining was negative for Ki-67 (Li: <10%) and P53 (-). These data indicated that the tumor was not of gastrointestinal and colonic origin.

Bilateral pleural effusion occurred on POD7 and was successfully treated with closed thoracic drainage. After being discharged, the patient underwent two cycles of chemotherapy combined with one cycle of radiotherapy and recovered well with no recurrence or metastasis.

## 3. Discussion

The most common tumors in the anterior mediastinum include germ cell tumors, thymic tumors and metastatic carcinoma. SRCC is a highly invasive cancer that is rarely found in the anterior mediastinum. The earliest related case dates back to 1989 which was called LECL.^[11]^ In this report, the tumor was surrounded by connective tissue containing polygonal epithelial cells, macrophages, lymphocytes and plasma cells. Also, remnants of the LECL could be seen at the edge of the tumor by optical microscopy. Although the tumor cells were rich in mucus and had signet ring cell-like morphology, the case was not diagnosed as SRCC because of its epithelial components in the LECL.

A previous study reported 10 cases of thymoma with signet ring cell-like morphology. However, the negative expression of intracellular mucin through periodic acid-Schiff staining and negative IHC results failed to support a potential diagnosis of SRCC. The observation of intracytoplasmic vacuoles may be due to intracytoplasmic lumina.

The first true case of anterior mediastinal SRCC was reported in 2018.^[12]^ A man with a huge anterior mediastinal mass was diagnosed with SRCC based on radiological and histopathological examinations. Signet ring cell-like morphology combined with positive IHC staining for CK7, CEA and CD5 further verified the thymic origin of the tumor. However, cytology alone and partial mass biopsy does not accurately represent the entire tumor mass. Also, another report described thymic SRCC with different IHC results reporting that the signet ring cell-like tumor cells were positive for CDX2 and CK20.^[13]^ postmortem pathology confirmed an accurate diagnosis of thymic SRCC. Enteric differentiation has also been confirmed in primary thymic adenocarcinoma^[14,15]^ with the expression of CDX2 and CK20 being observed outside of the digestive system tumors such as in cases of SRCC of the urinary bladder.^[16]^

In this study, we predicted that the tumor originated from a common mediastinal tumor. Routine preoperative examinations including laboratory tests, chest and abdominal CT of the chest, brain MRI, and whole-body bone ECT were undertaken. However, the elevated level of serum CA-125 indicated that the patient might have a gynecological or biliary tract disease; however, the pelvic and abdominal CT results were normal. Surprisingly, the postoperative pathological results of SRCC could not provide a definitive clinical diagnosis and so it was necessary to rule out other possible diseases.

Germ cell tumors, particularly tumors of the yolk sac tumor mostly occurred in young males and are commonly confused with anterior mediastinal tumors. The high levels of serum alpha-fetoprotein (AFP) and β-hCG and the presence of hyaline globules in cells can provide diagnostic information^[17]^ yet these were not observed in our case. The cell morphology and histopathology results along with the lack of LECL epithelial structure informed prevented a definitive diagnosis of LECL.

The recent discovery of primary lung SRCC^[10,18]^ raises the possibility of SRCC metastasis to the lung. However, IHC results for TTF-1, NapsinA and CK7 which are biomarkers for primary lung SRCC were negative in our case. Despite of the lack of mucin protein, the mucus components were significantly different from the intracytoplasmic lumina in the cytoplasm and the nuclei were abnormal which may suggest signet ring cell-like morphology. Moreover, positive results for CDX2, CK20 and SATB2 staining once cast doubt over the origin of the disease, however, the negative gastroenteroscopy, colonoscopy and abdominal CT ruled out gastrointestinal and biliary origins.

Thymic carcinoma with intestinal metaplasia appeared to be a plausible diagnosis but negative CD5 staining was observed in our case and it was not known if mild intestinal metaplasia of the stomach was related to the anterior mediastinal tumor. Furthermore, the high rate of positive cells for Ki-67 and a short follow-up period prevented us from ruling out other primary occult tumors. Longer follow-up and multiple whole-body radiological examinations could confirm the accurate origin of the anterior mediastinal tumor.

Besides that, the survival time of the patients did not exceed 1 month in all the reported cases, while our case has been followed up for five months without recurrence or metastasis. This proves that our treatment is effective.

In summary, we describe a rare case of anterior mediastinal SRCC but could not definitively conclude the origin of the tumor. This is the first reported case of an anterior mediastinal SRCC in which the patient survived for more than five months.

## Acknowledgements

The authors with no financial support for the research would like to thank all the reviewers who participated in the review, as well as MJEditor (www.mjeditor.com) for providing English editing services during the preparation of this manuscript.

## Author contributions

**Data curation:** Simin Liu.

**Formal analysis:** Anbang Zhao.

**Project administration:** Ming Mao.

**Supervision:** Ming Mao.

**Writing – original draft:** Anbang Zhao, Simin Liu.

**Writing – review &amp; editing:** Ming Mao.
